# 4-Ethyl­anilinium 4-methyl­benzene­sulfonate

**DOI:** 10.1107/S1600536810028047

**Published:** 2010-07-21

**Authors:** De-Hong Wu, Qi-Qi Wu

**Affiliations:** a College of Chemistry and Chemical Engineering, Southeast University, Nanjing 210096, People’s Republic of China

## Abstract

In the crystal structure of the title molecular salt, C_8_H_12_N^+^·C_7_H_7_O_3_S^−^, the 4-ethyl­anilinium cations and 4-methyl­benzene­sulfonate anions are linked into chains parallel to the *b* axis by inter­molecular N—H⋯O hydrogen bonds.

## Related literature

For background literature concerning mol­ecular–ionic compounds, see: Czupiński *et al.* (2002 ▶); Katrusiak & Szafrański (2006 ▶). For related structures. see: Chen (2009 ▶); Wang (2010 ▶).
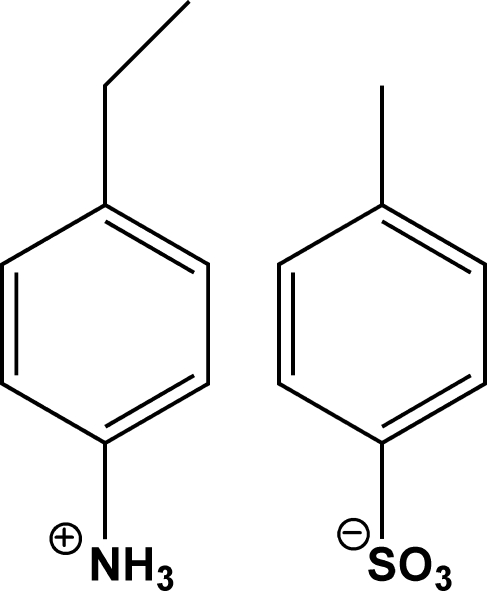


## Experimental

### 

#### Crystal data

C_8_H_12_N^+^·C_7_H_7_O_3_S^−^
*M* *_r_* = 293.38
Monoclinic, 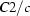

*a* = 25.016 (3) Å
*b* = 5.6376 (11) Å
*c* = 21.6387 (13) Å
β = 95.227 (10)°
*V* = 3039.0 (7) Å^3^
*Z* = 8
Mo *K*α radiation
μ = 0.22 mm^−1^
*T* = 291 K
0.36 × 0.28 × 0.24 mm

#### Data collection

Rigaku Mercury2 diffractometer
Absorption correction: multi-scan (*CrystalClear*; Rigaku, 2005 ▶) *T*_min_ = 0.930, *T*_max_ = 0.950
14572 measured reflections
3490 independent reflections
2614 reflections with *I* > 2σ(*I*)
*R*_int_ = 0.048

#### Refinement

*R*[*F* ^2^ > 2σ(*F* ^2^)] = 0.058
*wR*(*F* ^2^) = 0.178
*S* = 1.06
3490 reflections
181 parameters
H-atom parameters constrained
Δρ_max_ = 0.41 e Å^−3^
Δρ_min_ = −0.44 e Å^−3^

### 

Data collection: *CrystalClear* (Rigaku, 2005 ▶); cell refinement: *CrystalClear*; data reduction: *CrystalClear*; program(s) used to solve structure: *SHELXS97* (Sheldrick, 2008 ▶); program(s) used to refine structure: *SHELXL97* (Sheldrick, 2008 ▶); molecular graphics: *SHELXTL* (Sheldrick, 2008 ▶); software used to prepare material for publication: *SHELXTL*.

## Supplementary Material

Crystal structure: contains datablocks I, global. DOI: 10.1107/S1600536810028047/rz2476sup1.cif

Structure factors: contains datablocks I. DOI: 10.1107/S1600536810028047/rz2476Isup2.hkl

Additional supplementary materials:  crystallographic information; 3D view; checkCIF report

## Figures and Tables

**Table 1:** Hydrogen-bond geometry (Å, °)

*D*—H⋯*A*	*D*—H	H⋯*A*	*D*⋯*A*	*D*—H⋯*A*
N1—H1*D*⋯O1^i^	0.89	2.24	2.792 (3)	120
N1—H1*D*⋯O1	0.89	2.26	3.085 (3)	154
N1—H1*E*⋯O2^ii^	0.89	2.04	2.815 (3)	146
N1—H1*F*⋯O3^iii^	0.89	2.24	2.808 (3)	122

Symmetry codes: (i) 
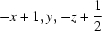
; (ii) 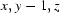; (iii) 
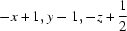
.

**Fig. 1.**
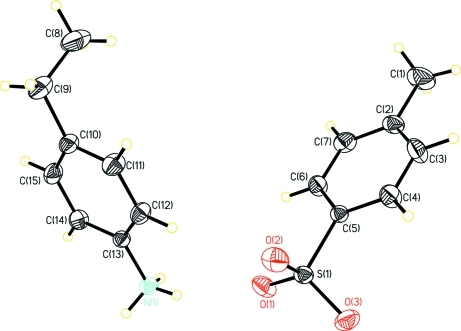
The molecular structure of the title compound, showing the atomic numbering scheme with 30% probability displacement ellipsoids.

**Fig. 2.**
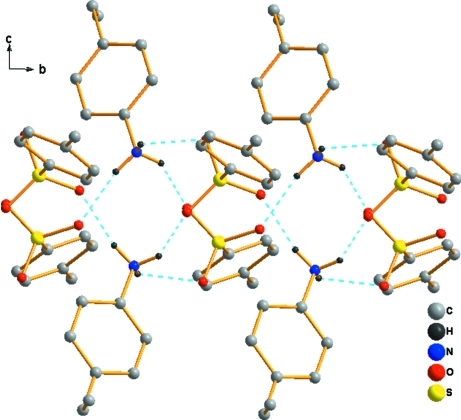
Crystal packing of the title compound viewed along the a axis. Hydrogen atoms not involved in hydrogen bonds (dashed lines) are omitted for clarity.

## supplementary crystallographic information

### Comment

Recently much attention has been devoted to simple molecular–ionic crystals containing organic cations and anions due to the tunability of their special structural features and their interesting physical properties (Czupiński *et al.*, 2002; Katrusiak & Szafrański, 2006). For similar structures, see: Chen, 2009; Wang, 2010. In our laboratory, the title compound has been synthesized and its crystal structure is herein reported.

The asymmetric unit of the title compound consists of a 4-ethylanilinium cation and a 4-methylbenzenesulfonate anion (Fig 1), in which proton transfer from the acid to the basic component has occurred (Fig. 1). In the crystal packing (Fig. 2), cations and anions are linked into one-dimensional chains parallel to *b*-axis by intermolecular N—H···O hydrogen bonds (Table 1).

### Experimental

4-Methylbenzenesulfonic acid hydrate (1.90 g; 10 mmol) was firstly dissolved in 50 ml ethanol, to which 4-ethybenzenamine (1.21 g; 10 mmol) was added under stirring at the ambient temperature to afford a clear solution without any participation. Single crystals suitable for X-ray structure analysis were obtained by slow evaporation of the above solution after 5 days in air (m. p. 192 °C).

The dielectric constant of the compound as a function of temperature indicates that the permittivity is basically temperature-independent (ε = C/(T–T_0_)), suggesting that this compound is not ferroelectric or there may be no distinct phase transition occurring within the measured temperature range between 93 K and 455 K.

### Refinement

H atoms were placed in calculated positions (N—H = 0.89 Å; C—H = 0.93 Å for C*sp^2^* atoms and C—H = 0.96 Å and 0.97 Å for C*sp^3^* atoms), assigned fixed *U*_iso_ values [*U*_iso_ = 1.2*U*_eq_(C*sp^2^*, N) and 1.5*U*_eq_(C*sp^3^*)] and allowed to ride.

### Crystal data

**Table:**

C_8_H_12_N^+^·C_7_H_7_O_3_S^−^	*F*(000) = 1248
*M**_r_* = 293.38	*D*_x_ = 1.283 Mg m^−^^3^
Monoclinic, *C*2/*c*	Melting point: 465 K
Hall symbol: -C 2yc	Mo *K*α radiation, λ = 0.71073 Å
*a* = 25.016 (3) Å	Cell parameters from 12307 reflections
*b* = 5.6376 (11) Å	θ = 3.1–27.6°
*c* = 21.6387 (13) Å	µ = 0.22 mm^−^^1^
β = 95.227 (10)°	*T* = 291 K
*V* = 3039.0 (7) Å^3^	Block, colourless
*Z* = 8	0.36 × 0.28 × 0.24 mm

### Data collection

**Table:**

Rigaku Mercury2 diffractometer	3490 independent reflections
Radiation source: fine-focus sealed tube	2614 reflections with *I* > 2σ(*I*)
graphite	*R*_int_ = 0.048
Detector resolution: 13.6612 pixels mm^-1^	θ_max_ = 27.5°, θ_min_ = 3.3°
CCD_Profile_fitting scans	*h* = −32→32
Absorption correction: multi-scan (*CrystalClear*; Rigaku, 2005)	*k* = −7→7
*T*_min_ = 0.930, *T*_max_ = 0.950	*l* = −27→28
14572 measured reflections	

### Refinement

**Table:**

Refinement on *F*^2^	Primary atom site location: structure-invariant direct methods
Least-squares matrix: full	Secondary atom site location: difference Fourier map
*R*[*F*^2^ > 2σ(*F*^2^)] = 0.058	Hydrogen site location: inferred from neighbouring sites
*wR*(*F*^2^) = 0.178	H-atom parameters constrained
*S* = 1.06	*w* = 1/[σ^2^(*F*_o_^2^) + (0.0988*P*)^2^ + 1.990*P*] where *P* = (*F*_o_^2^ + 2*F*_c_^2^)/3
3490 reflections	(Δ/σ)_max_ < 0.001
181 parameters	Δρ_max_ = 0.41 e Å^−^^3^
0 restraints	Δρ_min_ = −0.44 e Å^−^^3^

### Special details

**Table:**

Geometry. All e.s.d.'s (except the e.s.d. in the dihedral angle between two l.s. planes) are estimated using the full covariance matrix. The cell e.s.d.'s are taken into account individually in the estimation of e.s.d.'s in distances, angles and torsion angles; correlations between e.s.d.'s in cell parameters are only used when they are defined by crystal symmetry. An approximate (isotropic) treatment of cell e.s.d.'s is used for estimating e.s.d.'s involving l.s. planes.
Refinement. Refinement of *F*^2^ against ALL reflections. The weighted *R*-factor *wR* and goodness of fit *S* are based on *F*^2^, conventional *R*-factors *R* are based on *F*, with *F* set to zero for negative *F*^2^. The threshold expression of *F*^2^ > σ(*F*^2^) is used only for calculating *R*-factors(gt) *etc*. and is not relevant to the choice of reflections for refinement. *R*-factors based on *F*^2^ are statistically about twice as large as those based on *F*, and *R*- factors based on ALL data will be even larger.

### Fractional atomic coordinates and isotropic or equivalent isotropic displacement parameters (Å^2^)

**Table:**

	*x*	*y*	*z*	*U*_iso_*/*U*_eq_	
C1	0.18002 (12)	0.6568 (8)	0.1475 (2)	0.0771 (11)	
H1A	0.1636	0.7869	0.1243	0.116*	
H1B	0.1634	0.6386	0.1855	0.116*	
H1C	0.1754	0.5138	0.1235	0.116*	
C2	0.23944 (10)	0.7059 (5)	0.16208 (13)	0.0448 (7)	
C3	0.26270 (11)	0.9123 (6)	0.14269 (15)	0.0549 (8)	
H3A	0.2414	1.0231	0.1201	0.066*	
C4	0.31647 (11)	0.9576 (5)	0.15593 (14)	0.0471 (7)	
H4A	0.3311	1.0985	0.1428	0.056*	
C5	0.34871 (9)	0.7941 (4)	0.18873 (10)	0.0304 (5)	
C6	0.32643 (10)	0.5882 (5)	0.20886 (13)	0.0416 (6)	
H6A	0.3478	0.4769	0.2312	0.050*	
C7	0.27173 (11)	0.5477 (5)	0.19556 (13)	0.0473 (7)	
H7A	0.2568	0.4094	0.2098	0.057*	
C8	0.32058 (15)	0.5450 (11)	0.52795 (18)	0.1058 (18)	
H8A	0.3026	0.5895	0.5635	0.159*	
H8B	0.3130	0.3819	0.5180	0.159*	
H8C	0.3082	0.6432	0.4933	0.159*	
C9	0.37861 (13)	0.5763 (7)	0.54173 (14)	0.0620 (9)	
H9A	0.3908	0.4785	0.5771	0.074*	
H9B	0.3859	0.7404	0.5530	0.074*	
C10	0.41024 (10)	0.5113 (5)	0.48754 (12)	0.0414 (6)	
C11	0.41036 (12)	0.6573 (5)	0.43615 (13)	0.0473 (7)	
H11A	0.3919	0.8006	0.4358	0.057*	
C12	0.43700 (11)	0.5966 (5)	0.38534 (12)	0.0420 (6)	
H12A	0.4365	0.6969	0.3512	0.050*	
C13	0.46425 (9)	0.3857 (4)	0.38629 (11)	0.0317 (5)	
C14	0.46589 (10)	0.2380 (5)	0.43700 (12)	0.0417 (6)	
H14A	0.4851	0.0967	0.4375	0.050*	
C15	0.43868 (11)	0.3020 (5)	0.48717 (13)	0.0467 (7)	
H15A	0.4396	0.2019	0.5214	0.056*	
N1	0.49149 (8)	0.3138 (4)	0.33224 (9)	0.0373 (5)	
H1D	0.4874	0.4261	0.3033	0.056*	
H1E	0.4774	0.1786	0.3171	0.056*	
H1F	0.5263	0.2931	0.3435	0.056*	
O1	0.44138 (7)	0.6248 (4)	0.22484 (10)	0.0543 (6)	
O2	0.42345 (8)	1.0277 (4)	0.25272 (9)	0.0534 (5)	
O3	0.43719 (8)	0.9314 (4)	0.14712 (9)	0.0575 (6)	
S1	0.41818 (2)	0.84917 (11)	0.20463 (3)	0.0335 (2)	

### Atomic displacement parameters (Å^2^)

**Table:**

	*U*^11^	*U*^22^	*U*^33^	*U*^12^	*U*^13^	*U*^23^
C1	0.0355 (16)	0.105 (3)	0.089 (3)	−0.0119 (18)	−0.0011 (16)	−0.011 (2)
C2	0.0313 (13)	0.0591 (18)	0.0439 (14)	−0.0054 (12)	0.0019 (11)	−0.0108 (13)
C3	0.0425 (15)	0.0547 (18)	0.0650 (19)	0.0104 (13)	−0.0090 (14)	0.0114 (16)
C4	0.0420 (14)	0.0369 (14)	0.0616 (18)	−0.0030 (12)	0.0007 (12)	0.0127 (13)
C5	0.0289 (11)	0.0313 (12)	0.0311 (11)	−0.0021 (9)	0.0031 (9)	−0.0010 (9)
C6	0.0387 (13)	0.0366 (14)	0.0486 (15)	−0.0047 (11)	−0.0004 (11)	0.0118 (12)
C7	0.0450 (15)	0.0435 (16)	0.0543 (16)	−0.0164 (13)	0.0093 (12)	0.0019 (13)
C8	0.068 (2)	0.188 (5)	0.067 (2)	−0.008 (3)	0.032 (2)	−0.023 (3)
C9	0.069 (2)	0.076 (2)	0.0434 (16)	0.0024 (18)	0.0176 (15)	−0.0095 (17)
C10	0.0435 (14)	0.0475 (15)	0.0338 (13)	−0.0005 (12)	0.0067 (11)	−0.0056 (12)
C11	0.0578 (17)	0.0398 (15)	0.0454 (15)	0.0134 (13)	0.0117 (13)	−0.0001 (12)
C12	0.0542 (16)	0.0352 (14)	0.0373 (14)	0.0034 (12)	0.0084 (11)	0.0060 (11)
C13	0.0303 (11)	0.0342 (13)	0.0308 (12)	−0.0043 (10)	0.0037 (9)	−0.0019 (10)
C14	0.0441 (14)	0.0363 (14)	0.0449 (15)	0.0076 (12)	0.0045 (12)	0.0071 (12)
C15	0.0531 (16)	0.0511 (17)	0.0360 (14)	0.0065 (13)	0.0055 (12)	0.0122 (12)
N1	0.0373 (11)	0.0397 (12)	0.0355 (11)	−0.0024 (9)	0.0067 (8)	−0.0065 (9)
O1	0.0368 (10)	0.0510 (12)	0.0746 (15)	0.0090 (9)	0.0017 (9)	0.0067 (11)
O2	0.0467 (11)	0.0567 (13)	0.0561 (12)	−0.0120 (9)	0.0005 (9)	−0.0235 (10)
O3	0.0493 (12)	0.0805 (16)	0.0439 (11)	−0.0235 (11)	0.0106 (9)	0.0028 (11)
S1	0.0288 (3)	0.0368 (4)	0.0350 (3)	−0.0045 (2)	0.0031 (2)	−0.0028 (3)

### Geometric parameters (Å, °)

**Table:**

C1—C2	1.517 (4)	C9—H9A	0.9700
C1—H1A	0.9600	C9—H9B	0.9700
C1—H1B	0.9600	C10—C15	1.378 (4)
C1—H1C	0.9600	C10—C11	1.384 (4)
C2—C7	1.366 (4)	C11—C12	1.379 (4)
C2—C3	1.383 (4)	C11—H11A	0.9300
C3—C4	1.374 (4)	C12—C13	1.370 (3)
C3—H3A	0.9300	C12—H12A	0.9300
C4—C5	1.378 (3)	C13—C14	1.375 (3)
C4—H4A	0.9300	C13—N1	1.463 (3)
C5—C6	1.375 (3)	C14—C15	1.381 (4)
C5—S1	1.768 (2)	C14—H14A	0.9300
C6—C7	1.391 (4)	C15—H15A	0.9300
C6—H6A	0.9300	N1—H1D	0.8900
C7—H7A	0.9300	N1—H1E	0.8900
C8—C9	1.466 (5)	N1—H1F	0.8900
C8—H8A	0.9600	O1—S1	1.443 (2)
C8—H8B	0.9600	O2—S1	1.4451 (19)
C8—H8C	0.9600	O3—S1	1.448 (2)
C9—C10	1.518 (4)		
			
C2—C1—H1A	109.5	C8—C9—H9B	109.0
C2—C1—H1B	109.5	C10—C9—H9B	109.0
H1A—C1—H1B	109.5	H9A—C9—H9B	107.8
C2—C1—H1C	109.5	C15—C10—C11	117.7 (2)
H1A—C1—H1C	109.5	C15—C10—C9	121.1 (3)
H1B—C1—H1C	109.5	C11—C10—C9	121.1 (3)
C7—C2—C3	117.7 (2)	C12—C11—C10	122.0 (2)
C7—C2—C1	120.8 (3)	C12—C11—H11A	119.0
C3—C2—C1	121.5 (3)	C10—C11—H11A	119.0
C4—C3—C2	121.6 (3)	C13—C12—C11	118.6 (2)
C4—C3—H3A	119.2	C13—C12—H12A	120.7
C2—C3—H3A	119.2	C11—C12—H12A	120.7
C3—C4—C5	119.9 (3)	C12—C13—C14	121.1 (2)
C3—C4—H4A	120.0	C12—C13—N1	119.7 (2)
C5—C4—H4A	120.0	C14—C13—N1	119.2 (2)
C6—C5—C4	119.5 (2)	C13—C14—C15	119.2 (2)
C6—C5—S1	120.41 (19)	C13—C14—H14A	120.4
C4—C5—S1	120.08 (19)	C15—C14—H14A	120.4
C5—C6—C7	119.5 (2)	C10—C15—C14	121.3 (2)
C5—C6—H6A	120.2	C10—C15—H15A	119.3
C7—C6—H6A	120.2	C14—C15—H15A	119.3
C2—C7—C6	121.7 (3)	C13—N1—H1D	109.5
C2—C7—H7A	119.2	C13—N1—H1E	109.5
C6—C7—H7A	119.2	H1D—N1—H1E	109.5
C9—C8—H8A	109.5	C13—N1—H1F	109.5
C9—C8—H8B	109.5	H1D—N1—H1F	109.5
H8A—C8—H8B	109.5	H1E—N1—H1F	109.5
C9—C8—H8C	109.5	O1—S1—O2	112.62 (13)
H8A—C8—H8C	109.5	O1—S1—O3	112.74 (14)
H8B—C8—H8C	109.5	O2—S1—O3	112.34 (13)
C8—C9—C10	113.0 (3)	O1—S1—C5	105.43 (11)
C8—C9—H9A	109.0	O2—S1—C5	106.63 (11)
C10—C9—H9A	109.0	O3—S1—C5	106.43 (11)

### Hydrogen-bond geometry (Å, °)

**Table:**

*D*—H···*A*	*D*—H	H···*A*	*D*···*A*	*D*—H···*A*
N1—H1D···O1^i^	0.89	2.24	2.792 (3)	120
N1—H1D···O1	0.89	2.26	3.085 (3)	154
N1—H1E···O2^ii^	0.89	2.04	2.815 (3)	146
N1—H1F···O3^iii^	0.89	2.24	2.808 (3)	122

Symmetry codes: (i) −*x*+1, *y*, −*z*+1/2; (ii) *x*, *y*−1, *z*; (iii) −*x*+1, *y*−1, −*z*+1/2.
